# Treatment after anterior cruciate ligament injury: Panther Symposium ACL Treatment Consensus Group

**DOI:** 10.1007/s00167-020-06012-6

**Published:** 2020-05-09

**Authors:** Theresa Diermeier, Benjamin B. Rothrauff, Lars Engebretsen, Andrew D. Lynch, Olufemi R. Ayeni, Mark V. Paterno, John W. Xerogeanes, Freddie H. Fu, Jon Karlsson, Volker Musahl, Eleonor Svantesson, Eric Hamrin Senorski, Thomas Rauer, Sean J. Meredith, The Panther Symposium ACL Treatment Consensus Group

**Affiliations:** 1 Department of Orthopaedic Surgery, UPMC Freddie Fu Sports Medicine Center, University of Pittsburgh Medical Center, 3200 South Water Street, Pittsburgh, PA 15203 USA; 2 Department of Orthopaedic Sport Medicine, Technical University Munich, Munich, Germany; 3 Department of Orthopedic Surgery, Oslo University Hospital, Oslo, Norway; 4 Division of Orthopaedic Surgery, McMaster University, Hamilton, Canada; 5 Division of Sports Medicine, Cincinnati Children’s Hospital Medical Center, Cincinnati, USA; 6 Emory University School of Medicine, Brookhaven, GA USA; 7 Department of Orthopaedics, Institute of Clinical Sciences, Sahlgrenska Academy, University of Gothenburg, Gothenburg, Sweden; 8 Department of Trauma Surgery, University Hospital Zurich, Zurich, Switzerland; 9 Department of Orthopaedics, University of Maryland School of Medicine, Baltimore, MD USA

**Keywords:** ACL injury, ACL reconstruction, Non-operative treatment

## Abstract

Treatment strategies for anterior cruciate ligament (ACL) injuries continue to evolve. Evidence supporting best practice guidelines for the management of ACL injury is to a large extent based on studies with low-level evidence. An international consensus group of experts was convened to collaboratively advance toward consensus opinions regarding the best available evidence on operative vs. non-operative treatment for ACL injury. The purpose of this study is to report the consensus statements on operative vs. non-operative treatment of ACL injuries developed at the ACL Consensus Meeting Panther Symposium 2019. Sixty-six international experts on the management of ACL injuries, representing 18 countries, were convened and participated in a process based on the Delphi method of achieving consensus. Proposed consensus statements were drafted by the Scientific Organizing Committee and Session Chairs for the three working groups. Panel participants reviewed preliminary statements prior to the meeting and provided the initial agreement and comments on the statement via an online survey. During the meeting, discussion and debate occurred for each statement, after which a final vote was then held. Eighty percent agreement was defined a-priori as consensus. A total of 11 of 13 statements on operative v. non-operative treatment of ACL injury reached the consensus during the Symposium. Nine statements achieved unanimous support, two reached strong consensus, one did not achieve consensus, and one was removed due to redundancy in the information provided. In highly active patients engaged in jumping, cutting, and pivoting sports, early anatomic ACL reconstruction is recommended due to the high risk of secondary meniscus and cartilage injuries with delayed surgery, although a period of progressive rehabilitation to resolve impairments and improve neuromuscular function is recommended. For patients who seek to return to straight plane activities, non-operative treatment with structured, progressive rehabilitation is an acceptable treatment option. However, with persistent functional instability, or when episodes of giving way occur, anatomic ACL reconstruction is indicated. The consensus statements derived from international leaders in the field will assist clinicians in deciding between operative and non-operative treatments with patients after an ACL injury.

**Level of evidence** V.

## Introduction

Anterior cruciate ligament (ACL) injuries are one of the most common injuries of the knee, with an incidence of approximately 85 per 100 000 in patients aged between 16 and 39 years [26, 36, 49]. The ACL is the primary stabilizer of the knee limiting anterior tibial translation and internal rotation, with deficiency resulting in anterior and rotatory instability [54, 91]. The commonest mode of injury is a non-contact mechanism during pivoting, cutting, and jumping with the knee slightly flexed and in a valgus position [1, 5].

Both operative and non-operative treatments of an ACL injury continue to evolve [21, 22, 32, 78]. Improved understanding of the structure and function of the native ACL has supported the development and adoption of anatomic ACL reconstruction techniques [3]. In parallel, increased recognition of the resilience of the neuromuscular system in achieving dynamic, functional knee stability despite ACL deficiency has concurrently supported non-operative treatment as a viable strategy in some patients [13, 23].

Successful outcomes following both operative and non-operative treatment necessitate progressive rehabilitation, which entails staged and phase-adjusted physical therapy with the aim to address impairments, achieve functional stability, and to safely return to sport [64]. The acute phase after the injury or surgery focuses on the elimination of residual symptoms (effusion, pain) and impairments (range of motion, quadriceps activation, and strength). Subsequently, neuromuscular and perturbation training are implemented to improve knee stabilization [9, 19]. The last phase aims to further optimize muscular strength, return to pre-injury sports level through sport-specific exercises, and assess psychological readiness for the return to sport [3]. Any discussion of non-operative treatment within this consensus document implies the completion of a progressive, staged rehabilitation protocol.

Similarly, any discussion of operative treatment implies anatomic ACL reconstruction (Table 1), which intends to restore the ACL to its native dimensions, collagen orientation, and insertion sites [83]. Anatomic ACL reconstruction includes both single- and double-bundle techniques, followed by a progressive rehabilitation program that considers the natural healing cascade and ligamentization of the graft [65]. Following fixation during ACL reconstruction, biological graft transitions from a tendon to a structure with ultrastructural, biochemical, and mechanical properties more similar to the native ACL [74]. These properties of the graft depend on the phase of ligamentization, with the minimum graft strength occurring between 4 and 12 weeks postoperatively [65, 74]. Comprehensive rehabilitation after operative ACL reconstruction is also paramount for clinical outcome and return to sports.

**Table 1:** Anatomic ACL reconstruction checklist based on “evidence to support the interpretation and use of the anatomic anterior cruciate ligament reconstruction Checklist” [82]

1.	Individualization of surgery for each patient
2.	Use of 30 degree scope
3.	Use of an accessory medial portal
4.	Direct visualization of the femoral insertion site
5.	Measuring the femoral insertion site dimensions
6.	Visualizing the lateral intercondylar ridge
7.	Visualizing the lateral bifurcate ridge
8.	Placing the femoral tunnel(s) in the femoral ACL insertion site
9.	Transportal drilling
10.	Direct visualization of the tibial insertion site
11.	Measuring the tibial insertion site dimensions
12.	Placing the tibial tunnel(s) in the tibial ACL insertion site
13.	Femoral fixation
14.	Tibial fixation
15.	Knee flexion angle during femoral tunnel drilling
16.	Graft type
17.	Knee flexion angle during graft tensioning
18.	Documenting femoral tunnel position

Whereas operative treatment aims to reduce laxity, non-operative treatments aim to reduce functional instability and both thereby prevent further damage to the menisci and cartilage, which may contribute to post-traumatic osteoarthritis [58, 84]. Functional bracing, intended to reduce the risk of ACL injury by decreasing peak ligament strain, has not yet been conclusively shown to achieve this goal, as the evidence is still limited [29, 75].

There is still uncertainty as to which patients should undergo immediate surgery and which patients may be successfully treated non-operatively. Three different patient responses after ACL injury have been described: (1) a coper can return to the pre-injury level without surgery and subjective instability; (2) an adapter reduces his/her level of activity to avoid subjective instability; (3) a non-coper cannot return to pre-injury activity level due to subjective instability and episodes of giving way [61]. A screening tool to differentiate potential copers from non-copers was developed and included a combination of hop tests, questionnaires on general knee function, and the frequency of giving-way episodes [18, 60]. Patients categorized as potential copers thereafter participated in structured progressive rehabilitation with additional perturbation training [9, 19]. Regardless of this three-response concept, there is a strong historical view that the treatment approach should be determined through a shared decision-making process between the patient and the provider [8]. In particular, the physician should share information on the evidence-based treatment options while also considering the patient’s expectations and goals. While the patient and provider are the primary stakeholders in the shared decision-making process, the potential influence of secondary stakeholders, such as family and coaches, should be anticipated so as to minimize interests potentially conflicting with the health of the patient.

Taken as a whole, the current body of evidence regarding the treatment of ACL injury is to a large extent based on low level of evidence. Therefore, an international, multidisciplinary group of experts was assembled to develop expert- and evidence-based consensus statements to assist clinicians in managing this difficult pathology. The purpose of this article is to report the results of the consensus group addressing the best available evidence on operative vs. non-operative treatments of ACL injury that were developed at the 2019 Panther Symposium ACL consensus meeting.

## Materials and methods

An international and multidisciplinary group of experts of ACL injury, including orthopedic surgeons, sports medicine physicians, physical therapists, and scientists, were convened in a 1-year consensus-building effort, which culminated in the consensus meeting, at the University of Pittsburgh, PA, USA (Table 2). The symposium included experts from 18 countries, spanning six continents. Experts were assigned to one or more, of the three consensus groups defined by a specific subtopic within ACL injury. The operative vs. non-operative treatment consensus groups consisted of 34 participants. A modified Delphi method was used to develop the consensus statements.

**Table 2:**
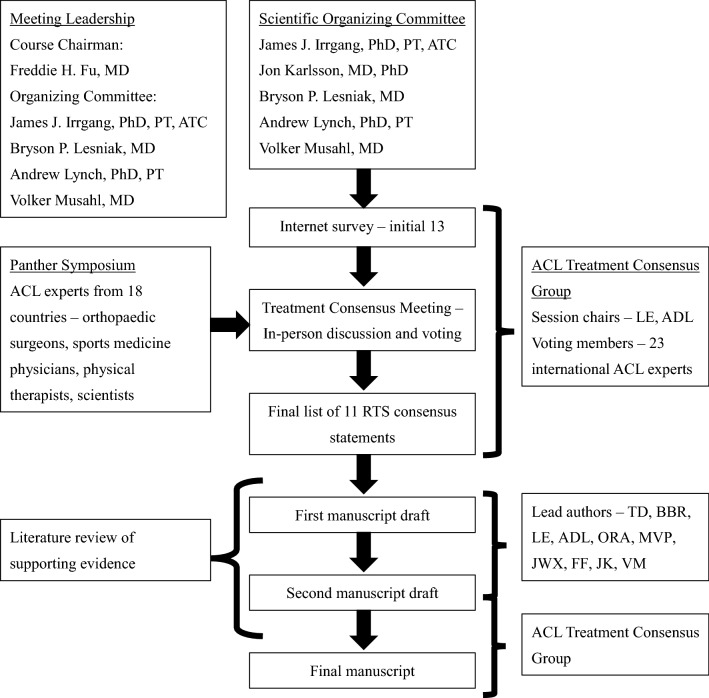
ACL Consensus Meeting Panther Symposium 2019

The scientific organizing committee and session chairs proposed a series of statements on the basis of a literature review. These were drafted with the aim of addressing areas of current controversy within the treatment of ACL injury, intended to assist clinicians in the management of this injury. Prior to the meeting, the proposed statements were presented to the panelists via a web-based survey. Each panelist indicated the extent of agreement or disagreement with each statement, and was asked to provide comments on each statement. On the third day of the 2019 Panther Symposium, after 2 days of presentations by symposium delegates on the current knowledge, a consensus discussion was held.

A total of 13 statements on the operative vs. non-operative treatment of ACL injury were discussed. The session was moderated by two experts (LE and ADL). Initial results and comments from the web-based survey were presented for each statement followed by discussion, debate, and revision by the working group. The consensus was determined by a show of hands. Satisfactory consensus was defined as 80% agreement. Opposing views were documented and discussed. Statements with less than 80% agreement were included in the consensus paper, noting the percentage of agreement. Statements felt to be irrelevant or redundant were excluded from this final paper.

This consensus group was assigned two liaisons (TD and BBR) who were responsible for amending each statement as requested over the course of the discussion. Liaisons transcribed the discussion and subsequently completed a literature review of MEDLINE for each finalized statement. To reduce the potential for bias in the data analysis and/or literature review, liaisons did not submit answers to the online questionnaire nor did they partake in the voting process.

## Results

Of the 13 statements discussed by this working group, nine achieved unanimous consensus, two achieved non-unanimous consensus, one did not achieve consensus, and one was excluded due to redundancy in the information provided (Table 3). The 12 finalized statements, with supporting literature, are as follows.

Operative and non-operative treatments are both acceptable treatment options for ACL injury.

Agree 23/23, 100%

**Table 3:** Consensus statements on non-operative and operative treatments of ACL injury

	Agreed statements	Agreement (%)
1	Operative and non-operative treatments are both acceptable treatment options for ACL injury	100
2	Operative versus non-operative treatments should be reached via a shared decision-making process that considers the patient’s presentation, goals, and expectations as well as a balanced presentation of the available evidence-based literature	82.6
3	The (injury) status of other stabilizing and supporting structures (e.g. meniscus, other ligaments, and cartilage) affects the decision to pursue operative or non-operative treatment	100
4	Individual anatomical differences (e.g., tibial slope, femoral morphology, alignment, etc.) may affect the stability of the knee after ACL injury and should be considered in the decision-making process for operative versus non-operative treatments	95.7
5	After an ACL injury, patients may be offered a period of progressive rehabilitation to improve impairments and improve overall function	100
6	An individual presenting with instability in their desired activity despite optimal rehabilitation should be referred for operative treatment	100
7	Development of osteoarthritis after an ACL injury is multifactorial and evidence is inconclusive following operative or non-operative treatments	100
8	In active patients wishing to return to jumping, cutting, and pivoting sports (e.g., soccer, football, handball, basketball): Operative treatment is the preferred option to maintain athletic participation in the medium-to-long term (1 to 5 + years after injury)	100
9	In active patients wishing to return to jumping, cutting, and pivoting sports (e.g., soccer, football, handball, basketball): Return to cutting and pivoting sports without surgery places the knee at risk of secondary injury (meniscus, cartilage, etc.)	100
11	In active patients wishing to return to straight plane activities (e.g., running, cycling, swimming, weight-lifting, etc.): Non-operative treatment is an option	100
12	In active patients wishing to return to straight plane activities (e.g., running, cycling, swimming, weight-lifting, etc.): In the case of persistent instability in daily life, operative treatment is appropriate for a return to non-rotational activities	100
	Not agreed statement	
10	In active patients wishing to return to cutting and pivoting sports (e.g., soccer, football, handball, basketball): Delayed operative treatment may be an option for temporary return to athletic participation following non-operative treatment accepting the risk of additional injury	43.4

After ACL injury, some patients are able to regain good functional knee stability following non-operative treatment entailing progressive rehabilitation and are able to return to pre-injury sports activity level without an ACL reconstruction (copers) [27, 28], but the identification of these patients has been challenging [80]. In a prospective study, the combination of hop tests, muscle strength, subjective instability (episodes of giving way), and knee function was found to be a moderate predictive tool for the identification of potential copers [18, 28, 32, 60]. A randomized-controlled trial comparing operative and non-operative treatments in 121 young active, non-elite patients with isolated ACL tears demonstrated no superiority of either treatment with regard to patient-reported outcomes at 2- and 5-year follow-up [21, 22]. However, almost 40% of the patients who were initially assigned to the non-operative treatment group required delayed ACL reconstruction and 32% of the patients (29 menisci in 19 patients) had subsequent surgery for meniscal pathology during the 2 year follow-up period. In contrast, 34 patients (56%) who underwent the early ACL reconstruction also had meniscus treatment (24 partial resection and 10 fixation) simultaneous with the ACL reconstruction, but only 10% (6 meniscal injuries in 5 patients) in the operatively treated group had meniscal injuries that required surgical treatment during follow-up [21]. With regard to knee laxity, as measured by KT-1000 and pivot shift test, non-operative treatment resulted in a larger anterior tibial translation (9.0 mm vs. 6.6 mm) and higher rate of rotatory laxity (positive pivot shift test: 78% vs. 25%). A matched-paired study based on the Swedish National ACL registry comparing operative and non-operative treatments after ACL injury reported superior results for quality of life, knee function, and symptoms at 1, 2, and 5 year follow-up for ACL reconstruction compared with non-operative treatment [40]. Another prospective trial with highly active patients included 832 patients at baseline with sub-acute ACL tear, whereas 345 patients were initially screened for the possibility of non-operative treatment. Based on the results of various hop tests, subjective instability, and general knee function, 146 patients were classified as potential copers, and at the final follow-up after 10 years, only 25 patients had not undergone ACL reconstruction [32].

Conclusion: Operative and non-operative are both acceptable treatment options after ACL injury, and a decision based on concomitant injuries, risk factors, level of activity, and patient’s expectations and goals is recommended as demonstrated in the following statements.

Operative versus non-operative treatment should be reached via a shared decision-making process that considers the patient’s presentation, goals, and expectations as well as a balanced presentation of the available evidence-based literature.

Agree 19/23, 82.6%

Before a particular treatment approach is pursued, the provider (physician and/or physical therapist) should present the evidence for operative and non-operative treatment options for an ACL injury to the patient. Based on the patient’s activity level, goals, and expectations, a decision should be made with the patient (and parents/guardians for minors) and provider as the primary stakeholders [8]. Physicians and physical therapists must be aware that personal and situational factors, such as level of competition, time in season, playing status, and role in the team, could affect the injured athlete's treatment decision. Parents and coaches are often the first individuals from whom athletes seek support or advice [59]. However, the coach may be conflicted by the interests of the team and the athlete’s immediate and future health [20, 33]. For some athletes, reactions and comments of parents related to the athlete’s injury were reported to negatively affect the athlete's treatment decision, with pressure to return to sport [59]. Due to the possible conflict of interest, secondary stakeholders such as family, coaches, and agents, among others, should not be directly involved in the decision-making process, although their indirect involvement may be considered.

Conclusion: Shared decision-making of the treatment option should be based on the evidence for operative and non-operative treatments, patient’s expectations and goals with the provider, and patient as the primary stakeholders.

The (injury) status of other stabilizing and supporting structures (e.g. meniscus, other ligaments, cartilage) affects the decision to pursue operative or non-operative treatment.

Agree 23/23, 100%

**Fig. 1:**
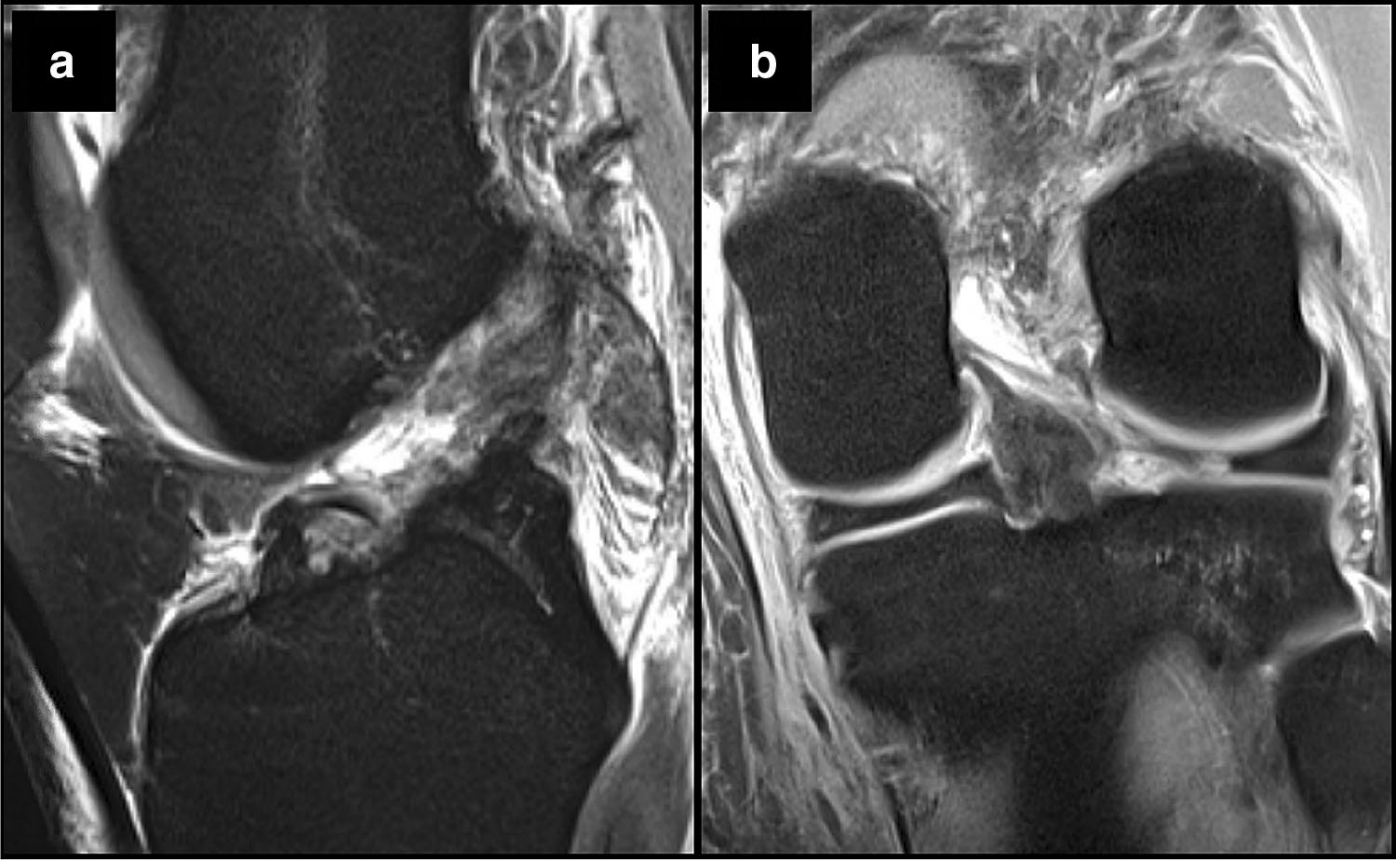
As seen in T2 MRI sequences, the patient sustained **a** complete ACL rupture and **b** associated lateral meniscus root tear

ACL injuries often occur together with concomitant injury to other knee structures, with meniscal injuries reported in 23–42%, cartilage lesions in 27%, and combined meniscal and chondral lesions in 15% of cases (Fig. 1) [6, 11, 41].

However, most studies investigating non-operative ACL treatment or studies comparing non-operative and operative treatment are limited to isolated ACL tears [21, 22, 32]. Based on clinical and biomechanical studies, an ACL reconstruction with concomitant meniscus repair may restore knee kinematics and results in improved patient-reported outcomes at short- and long-term follow-up [47, 69, 73, 90]. In contrast, simultaneously performed meniscectomy with ACL reconstruction is associated with poorer clinical outcomes, inferior knee kinematics, and a high rate (48–100%) of osteoarthritis in the long-term follow-up [12, 30, 50, 53, 89]. In case of delayed ACL reconstruction, a meniscectomy is more often performed than a meniscus repair [39]. The presence of concomitant knee injuries should, therefore, always be considered in the decision-making process, given the worse outcomes for meniscus injuries with delayed ACL reconstruction and higher rate of osteoarthritis in the long-term follow-up. In case of concomitant meniscus injury, anatomic ACL reconstruction with additional treatment of the meniscus injury is recommended.

In case of multiple ligament injuries involving the ACL and at least one other ligament, the literature has consistently demonstrated that operative management is superior to non-operative management [45, 66, 71]. Based on a recent systematic review, early (within 3 weeks after injury) reconstruction in a multiple ligament-injured knee was superior to delayed reconstruction with regard to clinical outcome measurements (Lysholm score, 90 vs. 82 out of 100 points) and resulted in higher rate of excellent/good IKDC scores (47% vs. 31%) [45]. Although failure after ligament reconstruction is not consistently defined in the literature (i.e., the need for revision vs. objective laxity vs. re-rupture on imaging vs. KOOS score < 44), the failure rate in a multiple ligament-injured knee is lower for reconstruction (6–9%) compared with repair techniques (37–40%) [44, 78].

Conclusion: The presence of a repairable meniscal lesion or a multiple ligament injury is an indication for an early anatomic ACL reconstruction with concomitant treatment of the other injured structures (meniscus repair and ligament repair/augmentation).

Individual anatomical differences (e.g., tibial slope, femoral morphology, alignment, etc.) may affect the stability of the knee after ACL injury and should be considered in the decision-making process for operative versus non-operative treatment.

Agree 22/23, 95.7%

**Fig. 2:**
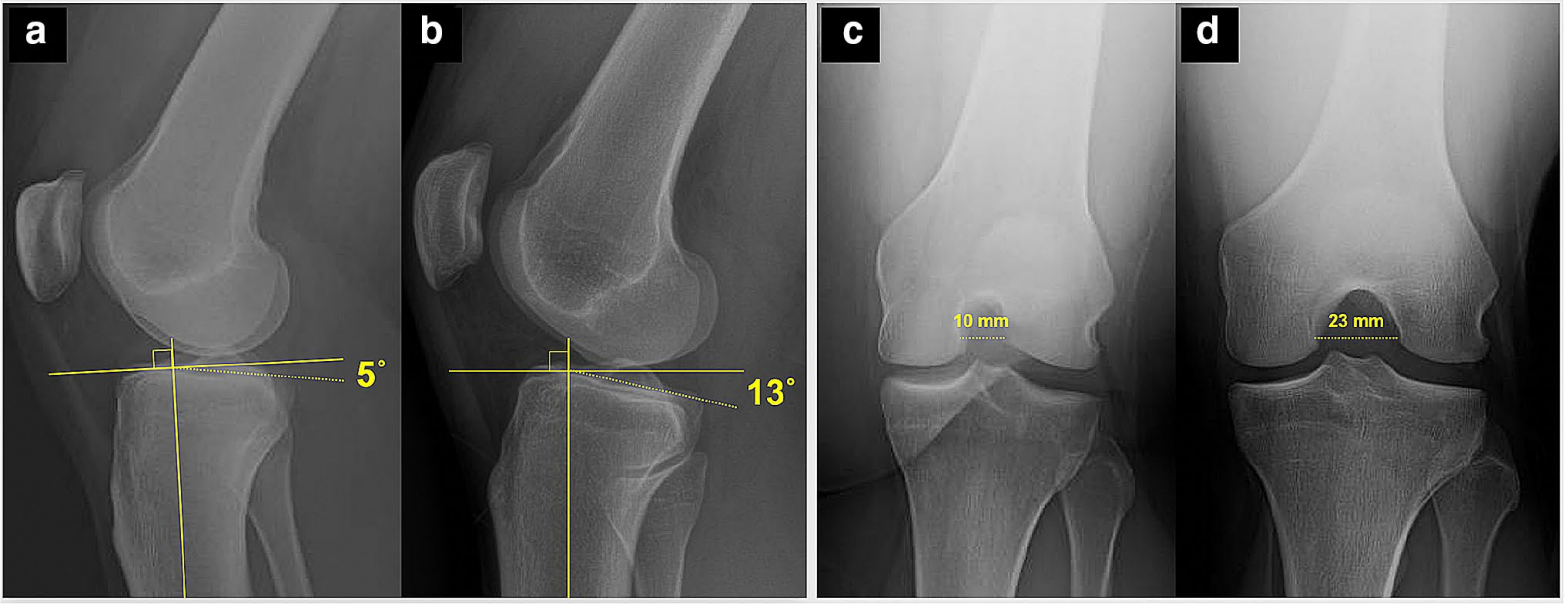
**a**, **b** Posterior tibial slope varies among patients, with greater slope increasing the risk of failure following ACL reconstruction. **c**, **d** Notch dimensions vary among patients, with small notch width dimensions constituting a relative contraindication for double-bundle ACLR

Bony morphology and soft tissue injury patterns have been demonstrated to influence knee joint laxity. An increased posterior tibial slope is associated with increased anterior tibial translation, as well as with increased rotatory instability (Fig. 2) [70, 86]. In addition, an increased lateral femoral condyle ratio resulted in increased rotatory instability [67, 68]. Severe varus limb alignment (> 5°) was demonstrated to increase the risk for more rapid degeneration of the medial compartment in the ACL-deficient knee, and is also a risk factor for secondary failure after an ACL reconstruction [34, 62]. Whereas lateral meniscus tears and a complete lateral meniscectomy result in increased rotatory instability [31, 55], a complete medial meniscectomy more strongly affects anterior tibial translation. However, general joint laxity (Beighton hypermobility score > 4) is not associated with increased rotatory laxity in the ACL-deficient knee [79].

Conclusion: Bony morphology features (increased posterior tibial slope, severe varus limb alignment, etc.) and concomitant injuries associated with increased or persistent knee instability should be considered in the decision-making process and are a relative indication for operative treatment.

After an ACL injury, patients may be offered a period of progressive rehabilitation to improve impairments and improve overall function.

Agree 23/23, 100%

Knee joint effusion, limited range of motion, and decreased quadriceps strength in the injured leg are common impairments initially after an ACL injury [10, 48]. Effusion can limit quadriceps function and in turn affect knee joint mechanics [63]. Progressive rehabilitation is useful in treating these initial impairments [32]. In patients with the possibility of non-operative treatment (absence of concomitant meniscus injuries or multi-ligament injuries requiring surgical treatment) before the evaluation of knee instability, a phase of rehabilitation is recommended to treat the initial impairments. Afterward, evaluation by hop tests, assessment of strength, overall knee function, and subjective instability are recommended to quantify the patient's potential for non-operative treatment. If progressive rehabilitation does not provide a satisfactory outcome, then operative intervention needs to be pursued and the progressive rehabilitation will have enhanced the post-surgical outcome [14]. In a cohort study with 2,187 patients after the resolution of impairments, one group was treated with neuromuscular training (i.e., strengthening and neuromuscular training) before ACL reconstruction and was compared to immediate ACL reconstruction. At 2-year follow-up, preoperative progressive rehabilitation before ACL reconstruction resulted in better patient-reported outcome (KOOS and IKDC), compared with ACL reconstruction without preoperative rehabilitation [14]. Whereas 63% of the patients without preoperative rehabilitation returned to sport at 2-year follow-up, which is similar to the reported rate (65%) in a meta-analysis from 2016, the rate increased to 72% in the group that completed preoperative rehabilitation [14].

Conclusion: Preoperative resolution of impairments and a period of rehabilitation is recommended for operative and non-operative treatments.

An individual presenting with instability in their desired activity despite optimal rehabilitation should be referred for operative treatment.

Agree 23/23, 100%

Persistent instability is a risk factor for further damage to the meniscus and cartilage [35]. Although the definitions of recurrent instability and episodes of instability vary in the current literature, a correlation between persistent and recurrent instability after ACL injury and meniscus and cartilage lesions has been demonstrated in several studies [2, 38, 77]. In a cohort study of 62 patients with acute ACL reconstructions, 37 with sub-acute ACL reconstructions, and 36 with chronic ACL reconstructions, one episode of giving way was associated with threefold higher odds for lateral meniscus tears. Timing of surgery and episodes of instability influenced the incidence of lateral meniscus tears with 1.45 higher odds in sub-acute (6–12 weeks) ACL reconstruction and 2.82 higher odds in chronic (> 12 weeks) ACL reconstruction [2]. Moreover, frequent episodes of instability are correlated with medial meniscus tears and chondral injuries [38]. Chondral defects and meniscectomy have been demonstrated as predictive factors for the development of osteoarthritis after ACL reconstruction [15, 37].

A partial ACL injury progressed to a complete ACL tear in 39% of young active patients treated non-operative, with half of the complete tears presenting with a concomitant meniscal lesion at the time of reconstruction. Age ≤ 20 years and participation in pivoting contact sports were identified as significant risk factors for progression to a complete tear [16].

Conclusion: If patient-reported instability or severe episodes of giving way occur during the progressive rehabilitation, patients should be referred for anatomic ACL reconstruction.

Development of osteoarthritis after an ACL injury is multifactorial and evidence is inconclusive following operative or non-operative treatment.

Agree 23/23, 100%

Osteoarthritis is the most common joint disease, affecting not only the cartilage, but all other tissues of the joint as well [24]. The pathomechanism of post-traumatic osteoarthritis (PTOA) has not been fully elucidated, but based on current research, the process of development of osteoarthritis is multifactorial [24]. Injuries, like ACL ruptures, can affect the joint biomechanics and cause chondral and meniscal lesions, and thereby reduce the sustainability of the joint. Matrix metalloproteases are responsible for cartilage destruction and synovial inflammation, and have been shown to be elevated following ACL injury and reconstruction [81, 85]. A meta-analysis of 24 observational studies found a fourfold increased risk for PTOA after knee injuries, although the definition of an injury was largely heterogeneous among the analyzed studies [56]. After ACL injury, the prevalence of PTOA is increased after both operative and non-operative treatments as compared to those without injury [17, 51, 58, 72]. Based on a recent systematic review with 41 included studies, the rate of OA after ACL reconstruction varied between 1 and 80%, with meniscectomy as the consistent risk factor for the development of OA [46]. Although long-term outcome studies after ACL reconstruction are available, the technique has evolved in the recent years, with a shift from non-anatomic ACL reconstruction to anatomic ACL reconstruction, limiting conclusions on the possible protective effect of anatomic ACL reconstruction.

Conclusion: Osteoarthritis after ACL injury is seen after both operative and non-operative treatments. Therefore, there is still a need for prospective, randomized-controlled trials to evaluate the hypothesized preventative effect of anatomic ACL reconstruction on the development of post-traumatic osteoarthritis.

In active patients wishing to return to jumping, cutting, and pivoting sports (e.g., soccer, football, handball, basketball):

Operative treatment is the preferred option to maintain athletic participation in the medium-to-long term (1–5+ years after injury).

Agree 23/23, 100%

In active patients wishing to return to pivoting and cutting sports, ACL reconstruction is the preferred treatment option to maintain participation in the medium-to-long term. However, overall, only 65% of patients return to their pre-injury sports level after ACL reconstruction and only 55% return to competitive level sport [4]. Although the exact reasons are still unknown, younger age, male gender, professional sports level, and positive psychological response were demonstrated to be associated with a successful return to pre-injury sports level after ACL reconstruction. In general, elite athletes return to their pre-injury level of sports after ACL reconstruction more often than recreational athletes [42, 88]. For instance, over 90% of elite soccer players were reported to return to the pre-injury level after ACL reconstruction [88]. Similarly, in a recent systematic review, the return to sport rate in elite football and basketball players was 78% and 82%, respectively [42]. In contrast, only 12.8% of high-level athletes returned to the pre-injury sports level with non-operative treatment, with a high rate of the secondary meniscus and cartilage damage; after 20 years, 95% of the patients underwent meniscectomy, during which 68% of patients were found to have chondral lesions [18, 57]. Overall, athletes returned to their pre-injury sports level between 6 and 13 months after ACL reconstruction [42].

Conclusion: In active patients, anatomic ACL reconstruction is the preferred treatment due to the higher rate of return to pre-injury sports level.

In active patients wishing to return to jumping, cutting and pivoting sports (e.g., soccer, football, handball, basketball):

Return to cutting and pivoting sports without surgery places the knee at risk of secondary injury (meniscus, cartilage, etc.).

Agree 23/23, 100%

In a prospective randomized-controlled trial, patients with high activity levels (median Tegner activity score of 9) with isolated ACL tears received the early operative treatment or non-operative treatment with the option of delayed ACL reconstruction. Although no differences were evident for patient-reported outcomes, at 2-year follow-up, patients in the “optional” operative treatment group had more self-reported and clinical laxity of the involved knee and more meniscal surgery over a 5-year follow-up period [21]. In a separate cohort, the risk for sustaining at least one additional intra-articular injury increased by 0.6% with each month of delay in operative treatment [7]. The odds of secondary cartilage lesions increased by nearly 1% for each month of delay [25]. A delay in ACL reconstruction of at least 12 months almost doubled the risk for meniscal tears [7, 43]. Increased risk of secondary injury is especially noted in young (< 12 years) and skeletally immature patients [2].

Conclusion: Non-operative treatment increases the risk for secondary injuries if the patient wants to return to jumping, cutting and pivoting sports, due to the increased risk of further episodes of instability.

In active patients wishing to return to cutting and pivoting sports (e.g. soccer, football, handball, basketball):

Delayed operative treatment may be an option for temporary return to athletic participation following non-operative treatment accepting the risk of additional injury.

Agree 10/23, 43.4%

No consensus was reached for this statement. Some professional athletes and active patients want to delay ACL reconstruction to temporarily return to athletic participation (competition). Based on the current evidence, the risk of secondary damage to the knee (e.g., meniscus and cartilage) is high, especially in high-demand sports with jumping, cutting and pivoting. In a recent cross-sectional study, 860 patients were included with 47.2% being professional athletes. With regard to the prevalence of meniscus tears, medial, lateral, and combined lesions were found more often with increasing time from injury (TFI) to surgery (medial meniscus tear prevalence at 0–36-week TFI was 48.2% and when >  61 weeks was 59.3%). Not only did the prevalence of injury increase with time, the rate of meniscectomy also increased (medial meniscectomy at 0–36-week TFI was 7.5%, and when TFI was > 61 weeks, it was 12.8%) [76]

Conclusion: Delayed ACL reconstruction in active patients may be a treatment option, but the provider, as well as the patient, must be aware of the risk of secondary injuries with worse long-term outcomes.

In active patients wishing to return to straight plane activities (e.g., running, cycling, swimming, weight-lifting, etc.): Non-operative treatment is an option.

Agree 23/23, 100%

Straight plane activities are less demanding on the ligamentous stabilizers of the knee and, therefore, are amenable to non-operative treatment. The anteroposterior stability during straight plane activities might be maintained by muscular control, but coronal and rotational stability could not be compensated [87]. With specific neuromuscular training (perturbation training) additional to standard rehabilitation, unphysiological muscular co-contractions during walking can be minimized and normalized the knee kinematics in the ACL-deficient knee [9]. In a matched-paired study, non-operative treatment resulted in an earlier return (non-operative 3–4 months vs. operative 6–12 months) and a higher return to level II sports (non-operative 88.9% vs. operative 77.8%) as compared to operative treatment [28]. Another study demonstrated a significantly higher number of non-operative-treated patients returned to level II and level III sports compared to operative treatment [27].

Conclusion: For return to straight plane activities, non-operative treatment is an option.

In active patients wishing to return to straight plane activities (e.g., running, cycling, swimming, weight-lifting etc.):

In the case of persistent instability in daily life, operative treatment is appropriate for a return to non-rotational activities.

Agree 23/23, 100%

Straight plane activities are less demanding to the ligamentous stabilizers of the knee and are, therefore, amenable to non-operative treatment. If during the non-operative treatment, subjective instability persists or episodes of giving way occur, referral for consideration of anatomic ACL reconstruction is recommended [21, 52]. Moreover, the current evidence for the efficacy of non-operative treatment is limited to isolated ACL tears.

Conclusion: Based on the current evidence, persistent instability in activities of daily living is an indication for anatomic ACL reconstruction to restore knee laxity and prevent secondary injuries.

## Conclusion

The expert panel at the ACL Consensus Meeting Panther Symposium 2019 reached consensus, defined as > 80% agreement, on 11 of 12 statements in terms of operative vs. non-operative treatments for ACL injuries. Consensus was reached that both treatment options may be acceptable, depending on patient characteristics, including the type of sporting demands and the presence of concomitant injuries. In highly active patients engaged in jumping, cutting, and pivoting sports, the early anatomic ACL reconstruction is recommended due to the high risk of secondary meniscus and cartilage injuries with delayed surgery, although a period of progressive rehabilitation to resolve impairments and improve neuromuscular function may be recommended. For patients who want to return to straight plane activities, non-operative treatment with structured, progressive rehabilitation is an acceptable treatment option. However, with persistent functional instability, or episodes of giving way occur, anatomic ACL reconstruction is indicated.

Despite strong consensus by experts, there is a need for larger randomized trials with longer term follow-up in which the early surgery (followed by rehabilitation) is compared with a strategy of early rehabilitation and delayed surgery. There are insufficient data to guide treatment in instances when there are concomitant meniscal and collateral ligament injuries. Data on long-term clinical outcomes are needed to better understand the effect of ACL treatment of injuries, subsequent injuries to meniscus and cartilage, and the development of osteoarthritis.
