# Correction to: Treatment after anterior cruciate ligament injury: Panther Symposium ACL Treatment Consensus Group

**DOI:** 10.1007/s00167-020-06280-2

**Published:** 2020-09-25

**Authors:** Theresa Diermeier, Benjamin B. Rothrauff, Lars Engebretsen, Andrew D. Lynch, Olufemi R. Ayeni, Mark V. Paterno, John W. Xerogeanes, Freddie H. Fu, Jon Karlsson, Volker Musahl, Eleonor Svantesson, Eric Hamrin Senorski, Thomas Rauer, Sean J. Meredith, The Panther Symposium ACL Treatment Consensus Group, Olufemi R. Ayeni, Charles H. Brown, Terese L. Chmielewski, Mark Clatworthy, Stefano Della Villa, Theresa Diermeier, Lars Engebretsen, Lucio Ernlund, Christian Fink, Freddie H. Fu, Alan Getgood, Timothy E. Hewett, Yasuyuki Ishibashi, Darren L. Johnson, Jon Karlsson, Andrew D. Lynch, Jeffrey A. Macalena, Robert G. Marx, Jacques Menetrey, Sean J. Meredith, Volker Musahl, Kentaro Onishi, Mark V. Paterno, Thomas Rauer, Benjamin B. Rothrauff, Laura C. Schmitt, Romain Seil, Eric Hamrin Senorski, Rainer Siebold, Lynn Snyder-Mackler, Tim Spalding, Eleonor Svantesson, Kevin E. Wilk, John W. Xerogeanes

**Affiliations:** 1 Department of Orthopaedic Surgery, UPMC Freddie Fu Sports Medicine Center, University of Pittsburgh Medical Center, 3200 South Water Street, Pittsburgh, PA 15203 USA; 2 Department of Orthopaedic Sport Medicine, Technical University Munich, Munich, Germany; 3 Department of Orthopedic Surgery, Oslo University Hospital, Oslo, Norway; 4 Division of Orthopaedic Surgery, McMaster University, Hamilton, Canada; 5 Division of Sports Medicine, Cincinnati Children’s Hospital Medical Center, Cincinnati, USA; 6 Emory University School of Medicine, Brookhaven, GA USA; 7 Department of Orthopaedics, Institute of Clinical Sciences, Sahlgrenska Academy, University of Gothenburg, Gothenburg, Sweden; 8 Department of Trauma Surgery, University Hospital Zurich, Zurich, Switzerland; 9 Department of Orthopaedics, University of Maryland School of Medicine, Baltimore, MD USA

## Correction to: Knee Surgery, Sports Traumatology, Arthroscopy (2020) 28:2390–2402 10.1007/s00167-020-06012-6

The article “Treatment after anterior cruciate ligament injury: Panther Symposium ACL Treatment Consensus Group”, written by Theresa Diermeier, Benjamin B. Rothrauff, Lars Engebretsen, Andrew D. Lynch, Olufemi R. Ayeni, Mark V. Paterno, John W. Xerogeanes, Freddie H. Fu, Jon Karlsson, Volker Musahl, Eleonor Svantesson, Eric Hamrin Senorski, Thomas Rauer, Sean J. Meredith, The Panther Symposium ACL Treatment Consensus Group, was originally published Online First without Open Access. After publication in volume 28, issue 8, page 2390–2402 the author decided to opt for Open Choice and to make the article an Open Access publication. Therefore, the copyright of the article has been changed to © The Author(s) 2020 and the article is forthwith distributed under the terms of the Creative Commons Attribution 4.0 International License, which permits use, sharing, adaptation, distribution and reproduction in any medium or format, as long as you give appropriate credit to the original author(s) and the source, provide a link to the Creative Commons licence, and indicate if changes were made. The images or other third party material in this article are included in the article’s Creative Commons licence, unless indicated otherwise in a credit line to the material. If material is not included in the article’s Creative Commons licence and your intended use is not permitted by statutory regulation or exceeds the permitted use, you will need to obtain permission directly from the copyright holder. To view a copy of this licence, visit https://creativecommons.org/licenses/by/4.0.

